# Development and validation of the WebAd-Q Questionnaire to monitor adherence to HIV therapy

**DOI:** 10.11606/S1518-8787.2018052000337

**Published:** 2018-05-18

**Authors:** Felipe Campos Vale, Ernani Tiaraju de Santa-Helena, Maria Altenfelder Santos, Wania Maria do Espirito Santo Carvalho, Paulo Rossi Menezes, Caritas Relva Basso, Mariliza Henrique Silva, Ana Maroso Alves, Maria Ines Battistella Nemes

**Affiliations:** I Universidade de São Paulo. Faculdade de Medicina. Programa de Pós-Graduação em Medicina Preventiva. São Paulo, SP, Brasil; II Universidade Regional de Blumenau. Departamento de Medicina. Programa de Pós-Graduação em Saúde Coletiva. Blumenau, SC, Brasil; III Universidade de São Paulo. Faculdade de Medicina. São Paulo, SP, Brasil; IV Centro de Referência e Treinamento em DST e Aids do Estado de São Paulo. São Paulo, SP, Brasil

**Keywords:** Anti-HIV Agents, supply & distribution, HIV Infections, drug therapy, Medication Adherence, Surveys and Questionnaires, utilization, Validation Studies, Fármacos Anti-HIV, provisão & distribuição, Infecções por HIV, tratamento farmacológico, Adesão à Medicação, Inquéritos e Questionários, utilização, Estudos de Validação

## Abstract

**OBJECTIVE:**

To present the development and validation of the WebAd-Q Questionnaire, a self-report instrument to monitor adherence to antiretroviral therapy in HIV/AIDS centers in Brazil.

**METHODS:**

The WebAd-Q is an electronic questionnaire that has three questions about the use of antiretrovirals in the last week. It was constructed from interviews and focus groups with 38 patients. Its validity was tested in a study with a sample of 90 adult patients on antiretroviral therapy for at least three months. We used electronic monitoring bottles, pill counting, and self-report interview to compare adherence. The WebAd-Q was answered on the sixtieth day, twice, with at least one hour of interval. The viral load of the patients was obtained from the service records. We have analyzed the agreement between the answers to the WebAd-Q, the associations, and the correlations with viral load and performance compared to other measures of adherence.

**RESULTS:**

Among the invited patients, 74 (82.2%) answered the WebAd-Q. No difficulties were reported to answer the questionnaire. The average answer time was 5 min 47 sec. The set of three questions of the WebAd-Q obtained agreement of 89.8%, with Kappa of 0.77 (95%CI 0.61–0.94). The non-adherence answers of the WebAd-Q were associated with detectable viral load. We obtained moderate viral load correlations with the non-adherence scale according to the WebAd-Q. For the three questions of the WebAd-Q, patients with non-adherence answers were also reported as less adherent according to the other measures of adherence.

**CONCLUSIONS:**

The WebAd-Q answered all the issues considered relevant in the validation of questionnaires, was well understood by patients, was associated with viral load, and obtained good agreement and good performance compared to the other measures. The feasibility analysis of its implementation still depends on a national study on its applicability.

## RESUMO

**OBJETIVO:**

Apresentar o desenvolvimento e a validação do Questionário WebAd-Q, um instrumento de autorrelato para monitorar a adesão à terapia antirretroviral em serviços de HIV/Aids no Brasil.

**MÉTODOS:**

O WebAd-Q é um questionário eletrônico que contém três perguntas sobre a tomada dos antirretrovirais na última semana. Foi construído a partir de entrevistas e grupos focais com 38 pacientes. Sua validade foi testada em estudo com uma amostra de 90 pacientes maiores de 18 anos, sob terapia antirretroviral há pelo menos três meses. Foram utilizadas as seguintes medidas de adesão comparativas: monitoramento eletrônico, contagem de pílulas e entrevista de autorrelato. O WebAd-Q foi respondido no sexagésimo dia por duas vezes, com intervalo mínimo de uma hora. A carga viral dos pacientes foi obtida nos registros do serviço. Analisamos a concordância entre as respostas ao WebAd-Q, associações e correlações com a carga viral e o desempenho em comparação às demais medidas de adesão.

**RESULTADOS:**

Entre os pacientes convidados, 74 (82,2%) responderam ao WebAd-Q. Não foram relatadas dificuldades em responder ao questionário. O tempo médio de resposta foi de 5 min 47 seg. O conjunto das três questões do WebAd-Q obteve concordância de 89,8%, com Kappa de 0,77 (IC95% 0,61–0,94). As respostas de não adesão do WebAd-Q associaram-se à carga viral detectável. Foram obtidas correlações moderadas da carga viral com escala de não adesão segundo o WebAd-Q. Para as três perguntas do WebAd-Q, pacientes com respostas de não adesão foram também apontados como menos aderentes segundo as demais medidas de adesão.

**CONCLUSÕES:**

O WebAd-Q atendeu a todos os quesitos considerados relevantes na validação de questionários, foi bem entendido por pacientes, apresentou associação com a carga viral e obteve boa concordância e bom desempenho em comparação a medidas concorrentes. A análise da viabilidade de sua implementação ainda depende de um estudo nacional de aplicabilidade.

## INTRODUCTION

The success of antiretroviral therapy (HAART) for HIV/AIDS heavily depends on timely diagnosis^1^, adequate treatment, and high patient adherence to therapeutic regimens^2^.

In Brazil, among the estimated 781,000 persons living with HIV (PLHIV) in 2014, 649,000 (83.1%) were diagnosed. Among the diagnosed PLHIV, 405,000 (62.4%) received antiretroviral therapy from the Brazilian Unified Health System (SUS). Approximately 88% of the persons on HAART had an undetectable viral load^b^.

The current Brazilian clinical treatment protocol^a^ recommends the use of HAART in all adults with positive serology for HIV, even without immunological impairment^3^. Brazil adopts the 90-90-90 goal of UNAIDS, which proposes that countries should achieve by 2020: 90% of the estimated PLHIV diagnosed; 90% of the persons diagnosed on HAART; 90% of the persons on HAART with undetectable viral load^c^. Efforts to achieve these goals should progressively increase the number of persons on HAART in the country, which will require an expanded response from the SUS. Today, treatment is performed in approximately 971 SUS centers of different sizes and institutional configurations^4^ ^,^ ^5^.

In this context, in addition to the essential monitoring of adherence in the individual clinical follow-up^6^, it is urgent to treat adherence from a programmatic point of view^5^ ^,^ ^7^, that is, as an indicator of the performance of health centers. For health teams, the monitoring of the rate of adherence of patients is another proxy measure of the quality of care^8^ ^,^ ^9^, thus serving as an indicator of the outcome of interventions focused on promoting adherence^5^ ^,^ ^7^.

However, there is no consensus in the literature on a gold standard for measuring adherence to HAART. There are different methods of measurement, such as self-report, pill counting, pharmacy records, and electronic monitoring devices. Each method has advantages and disadvantages, which vary according to the context and the desired objectives^7^ ^,^ ^10^ ^,^ ^11^.

Self-reports as questionnaires structured for interviews or self-completion are commonly used to measure adherence, both in observational studies and in the collection of information from health centers, given their operational simplicity, low cost, and possibility to address several dimensions of adherence^12^.

In order to monitor adherence, self-report questionnaires must present an adequate balance between psychometric, theoretical, and pragmatic properties^13^. Psychometric properties are related to the reliability and accuracy of the measure, including the mitigation of information biases (such as memory or desirable response biases)^14^ ^,^ ^15^. These properties are usually tested by measures of validity, reliability, and acceptability. Although there is no standardization of the validation methods, questionnaires should generally consider validity in relation to a clinical outcome measure (construct validity) and in relation to other measures of adherence (criterion validity)^16^.

The theoretical properties involve the interpretation given to the measure and the adaptation of the instrument to its purpose. On the other hand, pragmatic properties are those that enable the implementation of the instrument: low cost, non-intrusive, can repeat the measure over time, self-applied, fast in the collection and organization of answers^13^.

In Brazil, the methods used to measure adherence to HAART are usually non-specific and dependent on the individual initiative of professionals. The lack of a standardized resource prevents the routine and homogeneous monitoring of the rates of adherence in health centers and hinders the evaluation of the results of activities aimed at improving adherence^17^. Frequently, only the clinical outcome is monitored by measuring the viral load, which, however, does not allow the detailing of what difficulties may be occurring in the intake of the medication. Although the medication given is accompanied by the Logistic Medication Control System (SICLOM), the use of this system is still predominantly operational and not always aimed at monitoring adherence. To date, there are no self-report questionnaires validated for routine use in SUS centers that assist PLHIV.

This study presents the development and validation of the web version of a self-report instrument to monitor the average rates of the adherence to HAART in health centers: the Web Adherence Questionnaire (WebAd-Q).

## METHODS

The WebAd-Q Questionnaire was developed and tested in a study performed between 2008 and 2010. The instrument was proposed based on literature review and the previous experience of the research team in assessing the adherence of persons living with HIV and other chronic diseases. Inspired by a self-report questionnaire previously developed by the team to measure adherence to drug therapy for hypertension^18^, the WebAd-Q has been specifically adapted to evaluate the adherence to HAART.

The WebAd-Q was developed to evaluate adherence at the collective level, that is, to provide average rates of adherence for management use in HIV/AIDS centers and in operational research studies.

The construction of the questionnaire sought to respect the following attributes: evaluate the main components of the act and the process of taking medication; have high sensitivity for non-adherence; mitigate the socially desirable response and memory biases; and be easily understandable and feasible for the routine services of the SUS. The answer to the questionnaire is anonymous, as the purpose of the questionnaire is not to evaluate individual adherence, but rather to allow health centers to periodically obtain the rates of adherence for all patients or groups of patients.

The WebAd-Q is designed to provide self-answers on a computer, accessible via the Internet^d^ or offline, containing multimedia resources and automatically generating a database of answers of restricted access. It is formatted as an interactive cartoon, which asks three questions about the use of medications in the last seven days. The texts were audio recorded so that the questionnaire could be both read and heard by the respondents. Based on ours instructions, a web designer produced three alternate designs for the images and screens, from which we chose two: one with a female image and one with a male image. The two drawings, as well as the writing of the questions, were analyzed in two phases by volunteer patients from two health centers with interviews in the waiting room and focus groups.

For this stage of development of the instrument, we defined an intentional sample of 38 patients, from both sexes, different social strata, education level, ethnic groups, and different therapeutic regimens, who are monitored at the STD/AIDS Reference and Training Center of the State of São Paulo (CRT-DST/AIDS-SP) and at the Clinic of Medical Specialties of São Bernardo do Campo (SAE-SBC). The operation of the answer system and the database was tested at CRT-DST/AIDS-SP.

Based on the consultations made, the research team instructed the final design and supported the web designer in the design of the application. The patients showed preference for the female version of the main character. The speeches of the characters were recorded by actors, guided by the research team.

The WebAd-Q begins with an introduction to the questionnaire made by the character “Coquetel”, which explains the procedures of the questionnaire and ensures the confidentiality of the participant. Then, the character “Silvia” appears, a patient who is in a health center where she went to get her medication. Silvia introduces herself, comments that sometimes she has difficulty taking her medication, and asks the respondent three questions: 1) “And you? In the last seven days, did you take any medication from your cocktail outside the time appointed by your doctor?”; 2) “In the last seven days, did you stop taking any medication from your cocktail?”; 3) “In the last seven days, did you take more or less pills from your cocktail?” (Figure).

**Figure:**
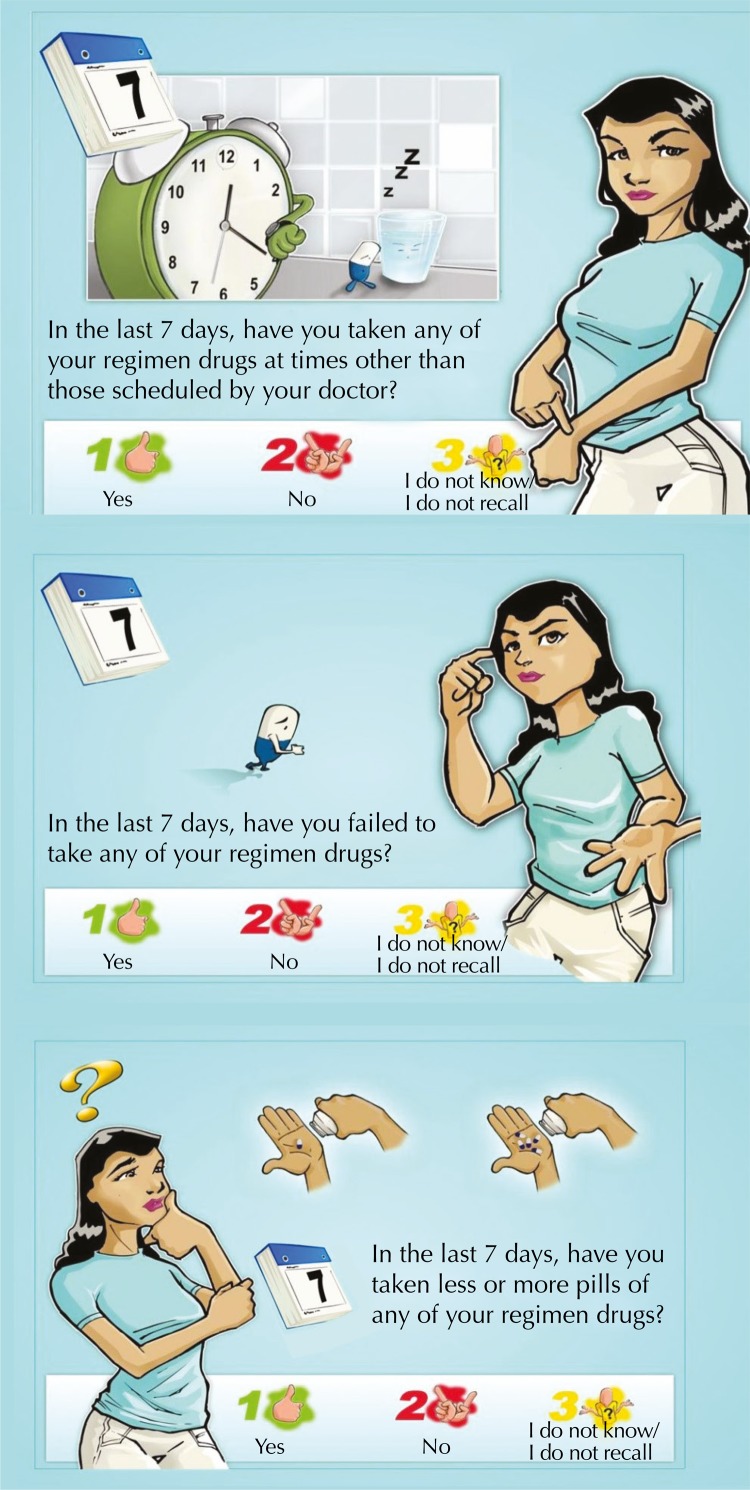
Screens of the questions of the WebAd-Q questionnaire with the character Silvia.

Possible answers for each question are: “No” (adherence answer), “Yes” or “I don’t know/I don’t remember” (non-adherence answers). In addition to the measures for each individual question, we constructed a scale of non-adherence of four categories, taking into account all three questions: 1) no non-adherence answer, 2) one non-adherence answer, 3) two non-adherence answers, and 4) three non-adherence answers.

For the validity study, we invited 90 adult patients on HAART for at least three months at the SAE-SBC, which is a health center that monitored approximately 800 persons on HAART at the time of data collection. The calculation of the sample size had sensitivity of 80%, precision of 10%, and prevalence of adherence of 70% as parameters. Patients were invited according to a convenience sample, according to the order of arrival in the center, from August to October 2009.

Patients who agreed to participate signed an informed consent and were instructed to use a special Medication Event Monitoring System (MEMS) bottle for 60 days, whose lid has a microprocessor that is activated when open, recording the date, time, and duration of the opening. Participants were asked to use the MEMS for the medication with the highest number of doses or pills of their antiretroviral prescription, as well as open the bottle only when taking the pills. They were also instructed to return to the health center after 30 and 60 days bringing the MEMS bottle.

The MEMS bottle allows us to construct two measures of adherence: MEMS dose of seven days (doses taken, calculated by the number of openings of the bottle in relation to the number of prescribed doses; we considered adherent patients with 100% of doses taken) and MEMS time of seven days (doses taken at the correct time, calculated by the number of openings of the bottle in a time interval of 15 minutes for more or less than prescribed in relation to the number of doses and time of the prescription; we considered adherent patients with 100% of doses taken at the correct time interval).

We also counted the pills given to the patients at the beginning of the study, after 30 days, and after 60 days. The patients were instructed to return on the thirtieth and sixtieth day with the pills not taken, recorded as “returned”. We made a percentage measure on non-adherence by the number of pills returned in relation to the number of pills supplied. The individual who returned 5% or more of the supplied pills was considered non-adherent.

On the thirtieth day, the participants reported their adherence to a trained interviewer using a previously tested self-report questionnaire^19^. After guidance, the patient was asked to recall the last three days and to identify the medication, time, and amount taken each day. We calculated the measure of adherence by the percentage of pills taken in relation to the total of pills prescribed. We considered adherent the individual who report taking 95% or more of the pills prescribed.

The results of the viral load of each patient were collected routinely in the SAE-SBC. In this center, viral load tests are requested from all patients in the follow-up every four months. For each patient, we analyzed the result of the viral load test closest to the answer to the WebAd-Q, collected before or after answering the questionnaire. As a mathematical resource to calculate the viral load log, we used the unit to express individuals with undetectable viral load.

For the analysis of the social, demographic, and clinical data of the participants, we calculated the absolute and relative frequencies and the respective 95% confidence intervals (95%CI) of the qualitative variables, and the medians, means, and respective standard deviations of the continuous variables.

The WebAd-Q was answered by the patients at the end of the sixtieth day of research, in a specific room for this purpose, after receiving instructions from previously trained health professionals. We asked patients to answer to the WebAd-Q twice, with a minimum interval of one hour, in order to estimate the test-retest agreement using the Kappa index. We calculated the results of the WebAd-Q and the other measures of adherence, with 95%CI.

In summary, on the thirtieth day, the patients performed electronic monitoring of doses and time, counted the pills, and answered the self-report questionnaire with the interviewer. On the sixtieth day, they again performed the electronic monitoring and pill counting and responded to the WebAd-Q. At the end of the study, we obtained the measure of viral load closest to the answer to the WebAd-Q, collected routinely in the center.

We tested the associations between detectable viral load and the answers to the WebAd-Q, with 95%CI (p < 0.05). We used the Spearman coefficient to analyze the correlation between the scale of non-adherence of the WebAd-Q and the viral load log, according to the viral load measure closest to the answer to the questionnaire. In addition to total correlation, we tested correlations for different groups of respondents, according to the distance between the date of the answer to the WebAd-Q and the date of viral load collection: up to seven days, from seven to 30 days, more than 30 days. We compared the answers for each question of the WebAd-Q with the other measures of adherence using the Mann-Whitney test.

## RESULTS

Of the 90 invited patients, 74 (82.2%) answered the WebAd-Q questionnaire. The average follow-up time in the study was 60.7 days [standard deviation (SD) of six days]. Among those who did not answer the questionnaire, there were two (2.2%) refusals and 14 (15.6%) drop-outs.

Table 1 presents the sociodemographic and clinical data of the patients who completed the study. We observed a higher proportion of male participants, mean age above 45 years, high proportion of nine years or more of education, long treatment time, reports of previous non-adherence and adverse reactions, and low proportion of persons who regularly use alcohol and drugs.

**Table 1:** Sociodemographic and clinical characteristics of the participants who completed the study on the validation of the WebAd-Q. (n = 74)

Variable	n, % or average	Standard deviation
Male	45	60.8
Average age (years)	45.6	11.2
Years of education^a^		
≤ 8	31	41.9
9–11	32	43.2
> 11	8	10.8
Average time of diagnosis (months)	113.3	59.2
Average time on HAART (months)^b^	94.9	55.1
Report of adverse reaction	53	71.6
Report of prior interruption of the HAART	25	33.8
Use of tobacco	23	31.1
Use of injectable drugs	7	9.5
Intake of alcoholic beverage		
Do not drink	32	43.2
Less than once a week	31	41.9
Weekly or more	11	14.9
Use of marijuana		
Do not use	71	95.9
Less than once a week	1	1.4
Weekly or more	2	2.7
Use of cocaine		
Do not use	70	94.6
Less than once a week	3	4.1
Weekly or more	1	1.4

WebAd-Q: Web Adherence Questionnaire; HAART: antiretroviral therapy

^a^ Excluded or ignored: n = 3 (4.1%).

^b^ Excluded or ignored: n = 1 (1.4%).

Among the 74 participants in the study, four (5.4%) reported difficulty in using MEMS, which resulted in the loss of information for MEMS dose and MEMS time in the last seven days. Two participants (2.7%) did not complete the self-report interview. Altogether, 17 patients (23.0%, 95%CI 13.4–32.6) had a detectable viral load (> 40 copies/mL) in the exam with the closest date.

Table 2 shows the measures of non-adherence obtained by the WebAd-Q questionnaire and the other methods. The analysis of all three questions of the WebAd-Q showed a total of 49 (66.2%; 95%CI 55.4–77.0) patients with one or more non-adherence answer. Regarding the measures for each question separately, the proportion of non-adherence was greater for question 1 (time). In relation to the non-adherence scale, we can observe decreasing proportions for each category of the scale, that is, as the number of non-adherence answers increases. In relation to the other measures, the proportion of non-adherence was higher for MEMS time, followed by MEMS dose, pill counting, and self-report.

**Table 2:** Results of non-adherence, according to the WebAd-Q and other methods.

Measure	Result		95%CI

	n	%	
Questions of the WebAd-Q (n = 74)			
Non-adherence to time (Q1)	46	62.2	51.1–73.2
Non-adherence to medication (Q2)	27	36.5	25.5–47.5
Non-adherence to dose (Q3)	11	14.9	6.8–23.0
Scale of non-adherence (n = 74)			
No non-adherence answer	25	33.8	23.0–44.6
1 non-adherence answer	21	28.4	18.1–38.7
2 non-adherence answers	21	28.4	18.1–38.7
3 non-adherence answers	7	9.5	2.8–16.1
Electronic monitoring (n = 70)			
MEMS of dose of 7 days	38	54.3	42.6–66.0
MEMS of time of 7 days	48	68.6	57.7–79.4
Pill counting (n = 74)			
PC of 30 days	24	32.4	21.8–43.1
PC of 60 days	23	31.1	20.5–41.6
Interview (n = 72)			
Self-report of 3 days	18	25.0	15.0–35.0

WebAd-Q: Web Adherence Questionnaire; PC: pill counting

The average time to answer the WebAd-Q questionnaire was 1 min 53 sec (SD = 1 min 53 sec). There was no statistically significant difference in the time to answer the questionnaire between patients with adherence or non-adherence answers, considering all three questions (p = 0.272). No patient reported difficulties in answering on the computer.

Of the total number of participants, 59 (79.7%) answered the questionnaire twice. In the test-retest, the answers for the set of three questions of the WebAd-Q obtained agreement of 89.8%, with Kappa of 0.77 (95%CI 0.61–0.94).

Table 3 shows the statistically significant associations between detectable viral load and non-adherence answers. The median distance between the answer to the WebAd-Q and viral load collection was 88 days, with an interquartile range of 91 days. The correlation between viral load log and non-adherence degree was moderate (r = 0.476, p < 0.001), with increasing trend according to the proximity of the date of viral load collection: r = 0.622 (p = 0.074) for a distance of up to seven days, r = 0.472 (p = 0.122) for a distance from seven to 30 days, and r = 0.428 (p = 0.001) for a distance greater than 30 days.

**Table 3:** Associations between detectable viral load and answers to the WebAd-Q. (n = 74)

Measure	n	Detectable viral load		OR (95%CI)	p

		n	%		
Q1 – Time					
Adherence	28	2	7.1	1.00	
Non-adherence	46	15	32.6	6.29 (1.32–30.08)	0.021
Q2 – Medication					
Adherence	47	4	8.5	1.00	
Non-adherence	27	13	48.1	9.98 (2.80–35.64)	< 0.001
Q3 – Dose					
Adherence	63	11	17.5	1.00	
Non-adherence	11	6	54.5	5.67 (1.47–21.96)	0.012

Total	74	17	23.0		

WebAd-Q: Web Adherence Questionnaire

Table 4 presents the comparison of the answers for each question of the WebAd-Q with the other measures of adherence. All measures were consistent with the answers obtained by the WebAd-Q. That is, in all measures used, when there was a statistically significant difference, the median adherence was higher for patients with adherence answers, according to the three questions of the WebAd-Q. Question 2 had the best performance, showing statistically significant differences between patients with adherence or non-adherence answers for all measures used, except for PC of 60 days.

**Table 4:** Comparison between the three questions of the WebAd-Q and the other measures of adherence.

Medidas	Q1 (time)					Q2 (medication)					Q3 (dose)				
		
	Adherence		Non-adherence		p	Adherence		Non-adherence		p	Adherence		Non-adherence		p
					
	median (min.; max.)	n	median (min.; max.)	n		median (min.; max.)	n	median (min.; max.)	n		median (min.; max.)	n	median (min.; max.)	n	
PC 30	100 (32.3; 107)	28	96.8 (52.2; 103.5)	46	0.010	100 (32.3; 107)	47	90.6 (52.2; 103.5)	27	0.003	98.9 (32.3; 107)	63	88.2 (56.3; 103.5)	11	0.349
PC 60	99.5 (64.4; 130.4)	28	97 (50.6; 125)	46	0.203	98.9 (50.6; 130.4)	47	96.9 (70.6; 125)	27	0.163	97.1 (64.4; 130.4)	63	100 (50.6; 123.2)	11	0.294
SR	100 (67; 200)	28	100 (17; 156)	44	0.073	100 (67; 200)	46	100 (17; 100)	26	0.019	100 (17; 200)	61	93.8 (17; 100)	11	0.006
MEMS D7	100 (43; 100)	28	89.5 (0; 100)	42	0.037	100 (0; 100)	46	86 (29; 100)	24	0.027	93 (29; 100)	60	100 (0; 100)	10	0.614
MEMS T7	93 (0; 100)	28	86 (0; 100)	42	0.055	92 (0; 100)	46	78.5 (29; 100)	24	0.028	86 (0; 100)	60	86 (0; 100)	10	0.891

WebAd-Q: Web Adherence Questionnaire; PC 30: pill counting of 30 days; PC 60: pill counting of 60 days; SR: self-report of 3 days; MEMS D7: electronic monitoring of the dose of 7 days; MEMS T7: electronic monitoring of intake time of 7 days

Mann-Whitney test.

## DISCUSSION

This study analyzed the validity of a self-administered questionnaire to measure adherence to antiretroviral therapy in care centers for PLHIV, and we observed high levels of test-retest agreement and good agreement between the answers and other indicators of adherence.

The associations between the answers to the WebAd-Q and viral load suggest consistency of the measures of the questionnaire for each question individually. The scale of non-adherence obtained by the WebAd-Q had moderate correlation when compared to the clinical outcome evaluated by the viral load, which is similar to that obtained by other self-report HIV questionnaires^20^ ^,^ ^21^. Furthermore, considering the increasing trend of this correlation according to the greater proximity of the answer to the date of viral load collection, the results suggest that the scale of non-adherence proposed is also theoretically consistent.

The scale seems to fit the purpose of the follow-up, as it provides health professionals with a numerical and synthetic measure of non-adherence for all patients, which helps in the identification of the most frequent types of non-adherence and provides subsidies for comparison, replanning, and evaluation of strategies.

This study used several measures of non-adherence, and it obtained different results for this measure, from 25% in the three-day self-report interview to 69% in the seven-day electronic monitoring measure that included time. This variability is expected, since different instruments evaluate different aspects of medication intake. The electronic monitoring records the opening of bottles and the pill counting records the number of pills not taken. The self-report records the pills taken according to the information given by the patient. All these methods are subject to reliability problems: the openings of the bottle may not match the pills taken, pills may be discarded, memory on the pills taken may fail, and the answer of the patient may be influenced by the interviewer or the environment^22^.

In a systematic review, Simoni et al.^21^ have shown that self-report questionnaires to measure adherence to HAART have their performance evaluated mainly by comparing them with the clinical outcome, measured by viral load: 78% of the studies included in this review compared the viral load, with significant associations in most cases, although without uniformity in the parameters used for the detection of viral load (detection limit). Approximately 35% of the studies compared other measures of adherence, such as electronic monitoring and pill counting. Only 22% compared viral load with another concurrent measure. One of the conclusions of the study is that the evaluations of self-report questionnaires lack methodological standardization.

Given this diversity, the validation of the WebAd-Q sought an expanded scope, based on the comparison with both viral load and different types of concurrent measure. Since there is no gold standard established and the comparison with the clinical outcome was satisfactory, we understand that the criterion (or concurrent) validity by comparison with other methods cannot be evaluated similarly as in studies on the accuracy of diagnostic tests. Thus, if the various methods evaluate different dimensions of adherence, we have to understand the comparison as an overall indicator of consistency between the measures obtained. This study showed that the groups of patients who provided non-adherence answers to the WebAd-Q were also considered less adherent by the other methods.

The questionnaire ensures the anonymity of the respondents, minimizing the desirable response bias. Although the research environment requires informed consent, the high non-adherence results obtained suggest that patients were comfortable to admit that they did not take or that they changed the dose of their medication.

The study showed the good pragmatic properties of the WebAd-Q: patients reported no difficulties in answering it; the average time to answer it was 5 min 47 sec, which is short considering that the questionnaire has an introduction and acknowledgments at the end; and the database is easily fed with answers, which dismisses the use of typists and can be consulted and shared online.

The purpose of the WebAd-Q was to develop a managerial instrument for health centers, with the provision of averages for the non-adherence of a group of patients in an autonomous, standardized, anonymous, and routine way. This would not be possible with an instrument that required a long time from patients and professionals, or if it were too complex to be answered and analyzed. That is why we chose the self-reported computer questionnaire, similar to the instruments used in other studies on adherence to HAART^15^ ^,^ ^23^.

The development of the instrument together with patients was fundamental in this aspect, since it ensured that respondents had friendly and interesting graphic elements and questions. These attributes were shown by several patients already in the first tests of the WebAd-Q in a large HIV/AIDS center, the CRT-DST/AIDS-SP, where a very diverse population answered the questionnaire in a “real-life” environment.

This study had limits regarding sample loss during the two months of the study, especially regarding the use of electronic monitoring bottles. Some of the drop-outs may also be related to difficulties in the use of MEMS, previously reported in the literature^24^. In future studies, prior training of participants to facilitate the handling of bottles could minimize the number of losses.

Although the rate of answers to the questionnaire remained above 82%, the decrease in sample number reduces the statistical power of the study. This may have hindered the observation of statistically significant results, particularly in relation to the comparison with other measures, which yielded some borderline results, but still enough for the criterion validity.

Another limit to be considered is that, in the context of the study, the concomitant use of different methods to measure adherence, together with viral load collection in the routine of the health center, may have generated greater attention and care from the patients in relation to the medication taken, reducing possible memory biases when answering the WebAd-Q.

Regarding the sociodemographic characteristics of the participants, we can observe the profile of users of a public health center in a medium-sized city in the state of São Paulo, which does not necessarily correspond to the profile of PLHIV in Brazil. For example, we can observe that the study participants presented a higher education level in relation to the average profile of Brazilians living with HIV, according to data from the last epidemiological bulletin on HIV/AIDS^e^.

In view of this limitation, the use of the WebAd-Q in the routine of the SUS was later tested in an applicability study conducted in a national sample of HIV centers from different regions of the country, which will be addressed in future publications^f^.

The WebAd-Q is a multidimensional questionnaire that includes taking all medications at the times and doses prescribed during the last week, with good reliability, patient friendly, and feasible for routine application in health centers. Therefore, in this validation study, the WebAd-Q showed adequate properties for the monitoring of the rates of non-adherence to HAART. The feasibility of its implementation in the routine of HIV centers will be analyzed in a national study, conducted after this study, which will seek to reflect the heterogeneity both of the persons under treatment in the country and the characteristics of the health centers that treat them.

## Footnotes

a Ministério da Saúde (BR), Secretaria de Vigilância em Saúde, Departamento de DST, Aids e Hepatites Virais. *Bol Epidemiol Aids DST*. 2015[cited 2016 Aug 11];4(1). Available from: http://www.aids.gov.br/pt-br/pub/2015/boletim-epidemiologico-hivaids-2015

b Ministério da Saúde (BR), Secretaria de Vigilância em Saúde, Departamento de DST, Aids e Hepatites Virais. Protocolo clínico e diretrizes terapêuticas para o manejo da infecção pelo HIV em adultos. Brasília (DF); 2013 [cited 2016 Aug 11]. Available from: http://www.aids.gov.br/system/tdf/pub/2013/64484/pcdt_adulto_270917.pdf?file=1&type=node&id=64484&force=1

c Joint United Nations Programme on HIV/AIDS. 90-90-90: an ambitious treatment target to help end the AIDS epidemic. Geneva: UNAIDS; 2014 [cited 2016 Aug 11]. Available from: http://www.unaids.org/sites/default/files/media_asset/90-90-90_en_0.pdf

d The questionnaire is available at: www2.fm.usp.br/webad-q. For access, the code “1999” must be used.

e Ministério da Saúde (BR), Secretaria de Vigilância em Saúde. HIV Aids 2017. *Bol Epidemiol Aids IST*. 2017 jan-jun [citado 31 jan 2018];5(1). Available from: http://www.aids.gov.br/system/tdf/pub/2017/65093/boletim_aids_internet_1.pdf?file=1&type=node&id=65093&force=1

f The applicability study resulted in the two doctoral theses listed below. The respective articles mentioned have been submitted for publication:
